# Life-stage niche partitioning and functional strategies promote predatory coccinellids’ co-occurrence

**DOI:** 10.1007/s00442-026-05866-w

**Published:** 2026-01-31

**Authors:** Ana Claudia da Silva, Débora P. Paula, David Andow, Patricia S. Sujii, Nícholas F. Camargo, Pedro H. B. Togni

**Affiliations:** 1 Programa de Pós-graduação em Ecologia, Instituto de Ciências Biológicas, Universidade de Brasília (UnB), Brasília, DF Brazil; 2 Embrapa Recursos Genéticos e Biotecnologia, Brasília, DF Brazil; 3 Department of Applied Ecology, North Carolina State University, Raleigh, NC USA; 4 Departamento de Ecologia, Instituto de Ciências Biológicas, Universidade de Brasília (UnB), Campus Darcy Ribeiro, Asa Norte, Brasília, DF 70910-900 Brazil

**Keywords:** Biodiversity, Competition, Density dependence, Generalist predators, Non-crop plants

## Abstract

Species coexistence is shaped by how individuals share limiting resources such as space, food, and shelter. Theory predicts that niche differentiation promotes coexistence, depending on habitat characteristics, behavioral traits, and the intensity of competition. Niche use may vary across life stages, as ontogenetic shifts alter habitat use and species interactions. We investigated habitat segregation, niche overlap, and the role of non-crop vegetation in affecting coccinellid coexistence across life stages through semi-controlled experiments and field sampling at 42 sites. Species showed consistent differences in habitat and microhabitat use across developmental stages, influenced by innate behaviors and plastic responses to interspecific interactions. Superior competitors (*Hippodamia convergens* and *Harmonia axyridis*) generally dominated prey-rich crop areas during egg, larval, and adult stages, but shifted to sheltered sites outside the plants during pupation. In contrast, *Eriopis connexa* used soil microhabitats throughout its life cycle, reducing niche overlap with other species. *Cycloneda sanguinea*, a competitively inferior species, persisted by exploiting non-crop plants, which increased spatial heterogeneity and resource availability. Non-crop vegetation thus promoted coexistence by enabling spatial segregation and reducing presumed competitive asymmetries across life stages. These patterns highlight how the strength of species interactions and spatial partitioning changes ontogenetically, reflecting both behavioral flexibility and the influence of habitat features. We propose a plastic functional classification of species based on their behavioral responses to potential competition-risk scenarios across life stages, ranging from risk-tolerant generalists to conditional risk-avoiders and niche-fidelity strategists, that could be broadened and adapted to other study systems.

**Supplementary Information:**

The online version contains supplementary material available at 10.1007/s00442-026-05866-w.

## Introduction

Understanding the influence of species interactions on community assembly has been a crucial goal in community ecology, given its central role in shaping community structure, diversity, and function (Paine 1966; Levine et al. 2017). However, the outcome of interactions on insect communities, for example, is influenced by life-history traits of interacting species (Lancaster et al. 2017), predation risk (Levine et al. 2017), habitat or microhabitat use throughout species life stages (Ingels and De Clercq 2011; Da Silva et al. 2022), temporal variation in resource availability, including seasonality and bi- or multivoltinism (Schoener 1972; Hood et al. 2021), and habitat structure (Pringle et al. 2019). Spatially heterogeneous environments shift the competitive abilities of holometabolous insects across their life stages, as they can exploit different microsites for foraging, oviposition, and pupation (Amarasekare 2003a).

In contrast, in spatially homogeneous environments, coexistence of competing species is possible if differences in species traits lead to niche differentiation (Amarasekare 2003a). In both scenarios, niche partitioning may stabilize coexistence when intraspecific competition outweighs interspecific competition, particularly when interspecific differences influence the latter in competitive ability, colonization capacity, and life-history trade-offs (Rocca et al. 2017). Such trade-offs are particularly relevant in guilds like aphidophagous coccinellids, where larval and adult stages rely mostly on prey (Sloggett 2008), females may compete for suitable oviposition sites, and individuals need to seek refuges for pupation (Hodek et al. 2012). As aphids exhibit an aggregated distribution, different species simultaneously exploit the same resources, and intense interspecific competition may arise (Hodek et al. 2012), thereby affecting their community composition and structure (e.g., Bahlai et al. 2015).

However, several traits have evolved within this guild to reduce interspecific competition, thereby shaping how they interact with habitat features through their life cycle (Evans 2003). Adults can assess patch quality using visual cues, herbivore-induced plant volatiles (Yi et al. 2023), plant constitutive volatiles (Togni et al. 2016), prey pheromones (Tapia et al. 2010), and con- and heterospecific semiochemicals (Hemptinne and Dixon 2000), including larval tracks (Seagraves 2009) and oviposition deterring pheromones (Evans 2003; Soares et al. 2025). When aphid densities are high, females select optimal oviposition distances based on the risk of egg predation near aphid colonies and the distance that prevents offspring starvation. This decision reflects the competition for ephemeral and aggregated resources and may be shaped by both active search and innate preferences (Evans 2003; Hodek et al. 2012; Togni et al. 2016; Soares et al. 2025).

Another important coccinellid behavior is the distribution of pupae across microsites, which may reflect competitive interactions during the larval stage. Larvae are less mobile than adults and frequently engage in intraguild predation and cannibalism (Lucas et al. 2000). The outcomes of these interactions are influenced by the size of the individuals (Yasuda et al. 2004), their behavior (Sato et al. 2009), competitive ability, such as aggressiveness (Yasuda et al. 2001), and the availability of spatial refuges (Da Silva et al. 2022). During the pupal stage, individuals are immobile and highly exposed to natural enemies, particularly parasitoids (Togni et al. 2015; Paula et al. 2021). As a result, the availability of safe pupation sites may become a critical resource at this stage. Although pupae aggregation associated with sunny surfaces has been observed (Honek et al. 2007), it has not been reported in tropical regions, where the exposure to solar radiation and high temperatures may pose a threat to pupae’ survival. In addition, aggregation of adults associated with their aposematic coloration was observed, particularly at overwintering sites (Wheeler et al. 2015; Honek et al. 2007), but not in tropical environments, where overwintering is not recorded for this group.

Coccinellid species usually coexist in the same habitat by modulating their spatial distribution within the plant (Rocca et al. 2022), or the inferior competitor may be displaced (Martins et al. 2009). Non-crop plants, typically native species that grow spontaneously within and around crops, can provide pollen, nectar, alternative prey, and shelter to aphidophagous coccinellids (Amaral et al. 2013; Venzon et al. 2019; Sicsú et al. 2020). While it is widely accepted that the coexistence of competing species requires some degree of niche differentiation (Amarasekare 2003a), it remains unclear whether the spatial distribution of coccinellids across life stages is primarily driven by innate responses to environmental cues or shaped by species interactions across life stages.

We hypothesized that the use of surrounding non-crop plants (habitat scale) and the spatial segregation within microhabitats (within the plant) across life stages allow both superior and inferior competitors to co-occur in the same area. The use of non-crop plants and spatial segregation of species across their life stages are likely driven by competitive interactions among species rather than by an innate response. To test these hypotheses, we evaluated how habitat and microhabitat use and niche overlap of aphidophagous coccinellids change across their life stages. Specifically, we evaluated: (a) how different life stages of coccinellid species segregate their habitat and microhabitat use; (b) the role of non-crop plants in mediating coccinellid species co-occurrence; (c) whether the niche overlap of coccinellid species changes across life stages.

## Materials and methods

### Insect rearing for cage experiments

We first conducted a cage experiment in an experimental field (see below) to evaluate microhabitat use by coccinellid species at distinct life stages in the absence of heterospecific competitors. We used only coccinellids reared in the laboratory for this experiment to obtain well-fed individuals of different species with similar ages, sizes, and environmental conditions. Field-collected adults of *Cycloneda sanguinea* (L.), *Eriopis connexa* (Germar), *Harmonia axyridis* (Pallas), and *Hippodamia convergens* Guerin-Méneville (Coleoptera: Coccinellidae) were reared in the laboratory (25 ± 2 °C, 70 ± 15% r.h., and 12 h of light) and provided with aphid ad libitum aphids (*Myzus persicae* Sulzer and *Lipaphis pseudobrassicae* Davis; Hemiptera: Aphididae), dilute honey, and water. These aphid species are common in organic crops in the region (Paula et al. 2021). We formed couples of the above-mentioned coccinellid species and kept them in individual plastic pots (500 ml) with food. After egg hatching, larvae were transferred to individual plastic pots (250 ml) and fed with the same diet as adults until pupation or use in the experiments.

We used 36-h molted fourth-instar larvae to evaluate microhabitat selection for pupation and recently mated adults to assess oviposition microhabitat selection. Recently hatched adults were kept individually and well fed for at least 7 days to increase the likelihood of successful mating. Forty-eight hours before setting up the experiment in the field, we formed couples and observed them until mating began. Couples were monitored until the male exhibited a body shaking behavior, which typically indicates sperm transfer and successful mating (Omkar and Srivastava 2002; Zeni et al. 2024).

### Cage experiment setup in the experimental field

The cage experiment was conducted between August and September 2019 at the Biology Experimental Station of the University of Brasília (15° 44′ S, 47° 53′ W), Federal District, Brazil. The average annual precipitation is 1500 mm, and the driest months are between May and September. The climate is type Aw according to the Köppen–Geiger classification (dry winters and hot humid summers) (Alvares et al. 2013). We conducted experiments during the driest period of the year, when relative humidity can fall below 15%, and when coccinellids and aphids are most abundant in the field.

Collards (var. *acephala*) were cultivated in an experimental area covering 450 m^2^ (10 m × 45 m), containing 30 plant rows with 10 plants per row (*n* = 300 plants). We kept the soil bare throughout the experiment by manually weeding it when necessary. Plants were spaced 0.5 m within the row and 1.5 m between plant rows. We obtained the seedlings from commercial growers. All plants were approximately 50 days old after seed emergence at the time of transplanting. The seedlings were manually transplanted to the field 1 month prior to the experiment to obtain uniformly sized plants. Soil preparation and fertilization were carried out in accordance with local technical recommendations. Plants were irrigated using a drip system.

Between 15 and 20 days before the experiments, we infested the collards with aphids (Hemiptera: Aphididae) of the species *Myzus persicae* or *Lipaphis pseudobrassicae*, collected in the field. We used these species because all coccinellids we evaluated feed on these aphids (Andow et al. 2023). We removed weeds, other aphid species, and other invertebrates as much as possible one day before the experiment began. To do so, we carefully inspected the plants and soil to remove the invertebrates, although we could not ensure that the plant was completely clean. After that, we covered the plants with voile fabric cages (1 m × 1 m). We maintained 150–200 aphids (mixed infestations of *M. persicae* and *L. pseudobrassicae*) per leaf to ensure that prey were not a limiting resource. The number of aphids per leaf was counted on alternate days in all previously infested plants until aphid populations reached the required densities for the experiments.

### Pupation and oviposition in the cage experiment

To assess microhabitat selection for pupation by coccinellid species, we released three to six fourth-instar larvae (depending on larvae availability) of the same species into each cage with a fine brush. These densities were selected based on pilot experiments that showed that larvae exhibited similar behaviors across these densities, due to plant architecture that reduced encounters within the plant. The larvae were placed directly on collard leaves, near aphid colonies, but on different leaves to prevent cannibalism or other competitive interactions. To evaluate oviposition microhabitat selection, single mated females of each species (see above) was released into a separate cage. They were placed on the leaves, near aphids, after mating in the laboratory. We set up 10 cages per species, each containing coccinellid larvae of a different species, for a total of 170 individuals: 44 *C. sanguinea*, 46 *E. connexa*, 40 *H. axyridis*, and 40 *H. convergens*. For the adult stage, we established 20 cages for *C. sanguinea*, 23 for *E. connexa*, 22 for *H. axyridis*, and 23 for *H. convergens*, totaling 88 adults observed throughout the experiment.

Each cage was observed daily (once a day) for three days. During the observations, we carefully inspected the cages to record the locations of egg clusters (i.e., clusters of five or more eggs) and pupae. The egg clusters (hereafter referred to as eggs) and pupae were carefully removed immediately after recording to document their exact locations. If eggs or pupae were found directly on the soil surface, at the plant base touching the soil, or on the cage basis in contact with the soil, these locations were categorized as “soil”. For eggs and pupae found on the collards, we divided the plant into three equal sections (lower, middle, and upper thirds) and recorded the positions of each. We also recorded the eggs and pupae on the cage walls.

On the final day of observation, we removed and thoroughly inspected the cages, including their edges and buried parts, for hidden eggs or pupae. We also examined soil cracks and dead leaves. The collards were then removed, and all plant parts were meticulously inspected. We also ensured that all leaves had aphids during the observations to guarantee that larvae and adults had sufficient prey throughout the experiment and were not foraging for limited food instead of selecting pupation and oviposition sites.

### Field sampling of coccinellids in organic farms

We conducted this study on 42 organic farms cropping brassicas (primarily collards) in the Brazilian Federal District (15° 46′ 13″ S, and 47° 44′ 46″ W) between 2020 and 2023, always from August to October (end of the dry season). Each farm was sampled once each year. These farms were owned by smallholder farmers, averaging 13.4 ± 7.1 ha (mean ± SE), which falls within the range of organic farm sizes in our study region (4–70 ha). Sampled farms were at least 2 km apart. All farms were certified as organic for a minimum of 5 years. They are required to comply with Brazilian organic farming legislation when implementing practices such as pest and weed management, disease control, and soil fertilization, thereby eliminating synthetic products from farm management (Togni et al. 2019).

The farms also featured vegetated field margins, which were usually used as living mulch. Although there were differences among farms, these non-crop areas were composed of diverse plant species but had comparable structural, compositional, and temporal resources. Non-crop plants were defined as those located outside the cropping area and not intentionally planted by farmers. Most were native ruderal species.

To evaluate how the coccinellids *C. sanguinea*, *E. connexa*, *H. axyridis*, and *H. convergens* partition their habitats and microhabitats in the field, we randomly sampled 1 m × 1 m quadrats in both the crop area and adjacent non-crop plant strips on each farm (*n* = 5 quadrats per habitat per farm). For non-crop plants, we sampled plant strips up to 5 m from the cropping area, a distance selected because farms had non-crop plant strips of varying sizes (5–10 m). At least two well-trained observers carefully inspected each quadrat, and all coccinellid life stages were manually collected. Sampling continued until no additional coccinellids were detected, with search times varying from 10 to 20 min, depending on vegetation cover within each plot.

We recorded species, life stage (egg, larva, pupa, or adult), habitat type (crop or non-crop area), and specific microhabitat location within the crop area. Microhabitats were classified in the cage experiments as: lower, middle, and upper plant thirds, and the soil. In the field sampling, we also recorded the life stages of coccinellids found in non-crop plants. Both mobile (larvae and adults) and immobile (eggs and pupae) stages were collected and transported to the laboratory for identification. Immobile individuals that could not be identified to the species level were counted and used only for analyses that did not require species identification. For eggs, we followed the same approach as in the cage experiments, considering only egg clusters with five or more eggs clustered in the same place (hereafter referred to as eggs). We assessed predator abundance by summing the number of individuals of each species and life stage (adults and larvae) across all quadrats within and outside the crop area, across all microhabitats, and then calculating abundance for each farm. We also counted the number of aphids on four plants per quadrat in the cultivated area (*n* = 40 per farm). The number of aphids was counted on three leaves of each plant. In non-crop plants, we recorded the frequency of aphid-infested plants, as aphids tended to dislodge during sampling. Aphid samples were taken to the laboratory for identification.

### Statistical analyses

To investigate whether coccinellid species differed in their use of habitat and microhabitat for oviposition and pupation sites in the cage experiments, we fitted two multinomial logistic regression models using the function *multinom* from the *nnet* package in R (Ripley et al. 2016). This type of model requires one outcome category(arbitrarily chosen, though typically the largest or a control group) to be set as the reference, with its regression coefficients fixed at zero to enable comparisons across the remaining categories (Ripley et al. 2016).

In the first model, the response variable was the microhabitat selected by each species for oviposition, and in the second model, the response variable was the microhabitat selected for pupation. In both models, the explanatory variable was predator species identity, with cages set as the reference category for sites, and *H. convergens* as the reference for species. We compared the models using the *anova* function to fit an Analysis of Deviance (ANODEV) with a Chi-squared test, contrasting the null model (without predictors) with the full model (including predator species identity) to assess whether the inclusion of predator species identity significantly improved the model’s ability to explain variation in the response variable.

To investigate whether coccinellid species differed in their habitat and microhabitat use in the field, we fitted a series of multinomial logistic regression models. In all models, the response variable was habitat and microhabitat used by coccinellids, while the main explanatory variable was predator species identity. We used non-crop plants as the reference category for sites and *H*. *convergens* as the species reference category in all models. Specifically, we tested the following models: a null model without predictors (model 1); a model including only coccinellid species (model 2); a model with additive effects of coccinellid species and mean coccinellid abundance per farm (model 3); a model including coccinellid species and its interaction with mean coccinellid abundance (model 4).

We also evaluated models assessing the potential effect of life stage: one with additive effects of coccinellid species and life stage (model 5) and another including their interaction (model 6). In addition, we tested a model incorporating coccinellid species and its interaction with mean coccinellid abundance, along with the additive effects of predator species and life stage (model 7). We included the interaction between predator species and mean coccinellid abundance (only larvae and adults) per farm to account for potential context-dependent effects on habitat and microhabitat selection. Model comparisons were conducted as previously described, and we selected model 4 because it had the lowest AICc and a simpler structure. For detailed model comparisons, see Table S1.

We used the Pianka niche overlap index to assess pairwise niche overlap among species. The index ranges from 0 (total separation) to 1 (complete overlap). We selected the Pianka index because it is a widely used metric for assessing mutual similarity in resource use among species. Although it is a symmetrical index, we use it here because it does not imply any a priori inference of directional dependencies, direct competition, or exclusion (Pianka 1974).

For the cage experiment, we calculated overlap separately for each life stage and by summing the total counts of eggs and pupae per species. In the field, we followed the same approach and summed the counts of larvae and adults. We combined life stages to evaluate interspecific differences for immobile and mobile stages, representing each species as a single ecological unit. For the field data on each farm, we calculated the indices only for larvae and adults, as these were the only life stages that could be identified across all farms.

We assessed differences in niche overlap among species pairs in the cage experiment using the Pianka index in a generalized linear mixed model (GLMM) with a beta distribution, implemented via the *glmmTMB* package (Brooks et al. 2017). To avoid exact zeros (Smithson and Verkuilen 2006), we adjusted index values by adding a small constant (ϵ = 1e−6). We treated the adjusted Pianka indices as the response variable, species pairs as fixed effects, and cages as random effects. For the field data, we applied the same approach, using farms as random effects. We tested group differences using model contrasts with the *emmeans* function and performed multiple comparisons with a Tukey post hoc test on the estimated marginal means and evaluated model assumptions using residual diagnostics from the *DHARMa* package (Hartig and Lohse 2022).

We assessed whether niche overlap among species and life stages differed from expectations under random assignment by comparing the observed mean Pianka index for species pairs with expected values from simulated null models. To reduce algorithmic bias, we performed 10,000 Monte Carlo permutations (Lehsten and Harmand 2006). We generated null assemblages for both the cage experiment (including all life stages combined, eggs only, or pupae only) and the field data (all stages combined, or eggs, larvae, pupae, or adults only). We used Lawlor’s RA3 algorithm to generate these randomized matrices, which assume that all species have equal probabilities of occupying any site, given their niche breadth (Lawlor 1980; Lehsten and Harmand 2006).

We randomized species-by-site associations in the resource-use matrix by shuffling species presence across sites (columns) while preserving each species’ total occurrences (rows) (Lawlor 1980). This approach maintained species-specific niche breadth (i.e., number of sites occupied) but randomized the specific sites used, disrupting any existing guild structure (Lawlor 1980). Although RA3 does not preserve site-level richness, we aimed to introduce some ecological realism to the model by preserving niche breadth (Gotelli and Ulrich 2012). We applied no additional constraints on species identities or the total number of sites. We performed all analyses in R (R Core Team 2025).

## Results

### Microhabitat use and niche overlap in the cage experiment

**Fig. 1:**
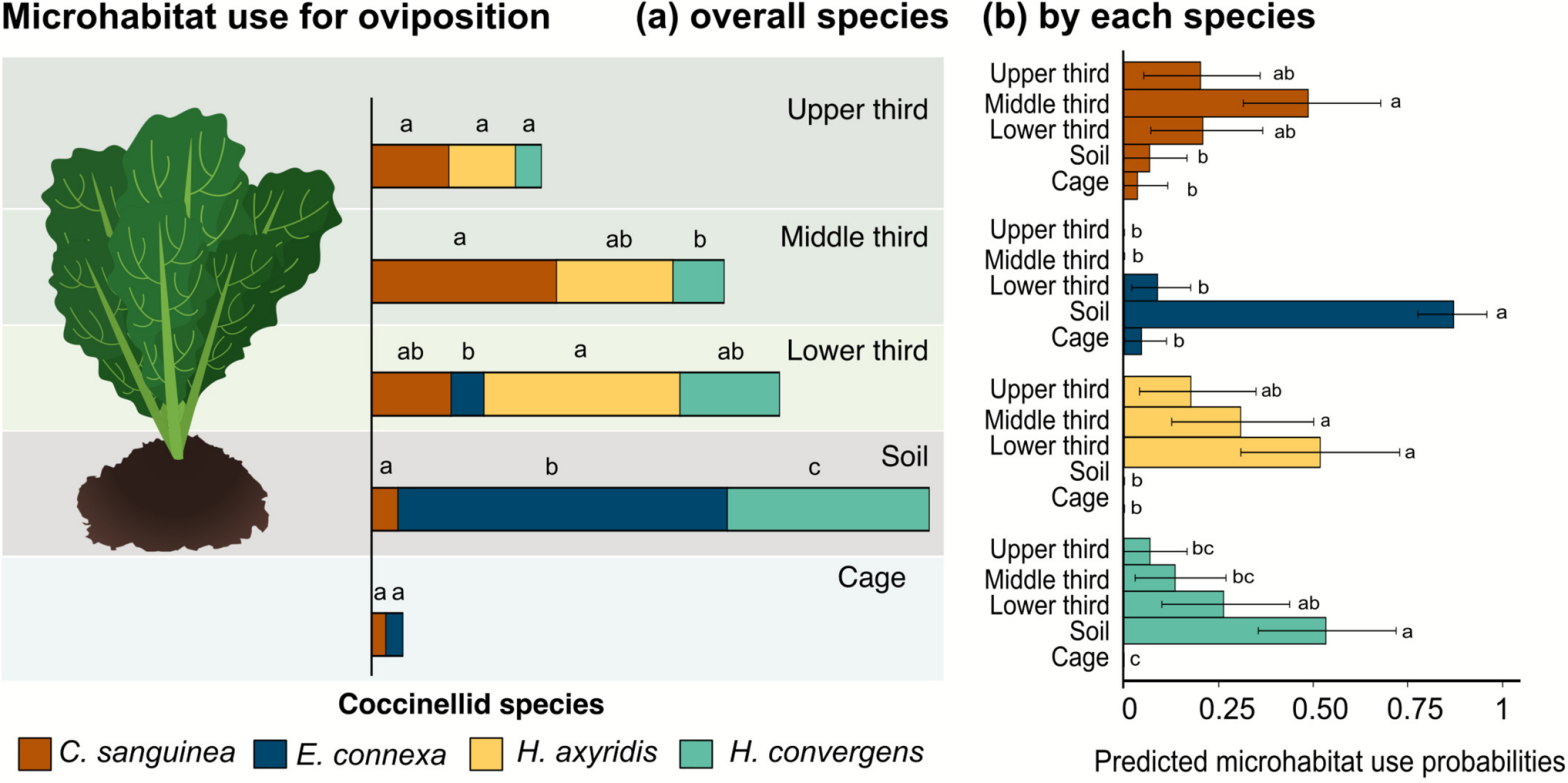
Predicted probabilities of microhabitat use for oviposition by overall coccinellid species (**a**) and by each species (**b**). Error bars represent 95% confidence intervals based on 1000 bootstrap resamples. Different letters indicate significant differences among species within the same microhabitat (**a**) and by each species across different microhabitats (**b**)

Microhabitat selection by adult coccinellids for oviposition varied among species (χ^2^ = 106.5; d.f. = 12; *p* < 0.001). The chances of *C*. *sanguinea* laying eggs in the middle third were greater than on the soil or cage. In contrast, *E*. *connexa* had a higher probability of ovipositing in the soil than in any other microhabitat. For *H*. *axyridis*, the likelihood of oviposition on the middle and lower thirds exceeded that on the cage and soil. In the case of *H*. *convergens*, the probability of laying eggs on the soil was higher than on the upper and middle thirds and on the cage; additionally, oviposition on the lower third was more likely than on the cage (Fig. 1).

*Cycloneda sanguinea* exhibited a greater tendency to oviposit on the middle third than *H*. *convergens*. On the lower third, *H*. *axyridis* had a higher probability of oviposition than *E*. *connexa*, whereas on the soil, *E*. *connexa* displayed the highest likelihood of laying eggs among all species. Still referring to the soil, *H*. *convergens* showed a higher probability of oviposition than *C*. *sanguinea* and *H. axyridis*, but still lower than *E*. *connexa* (Fig. 1).

**Fig. 2:**
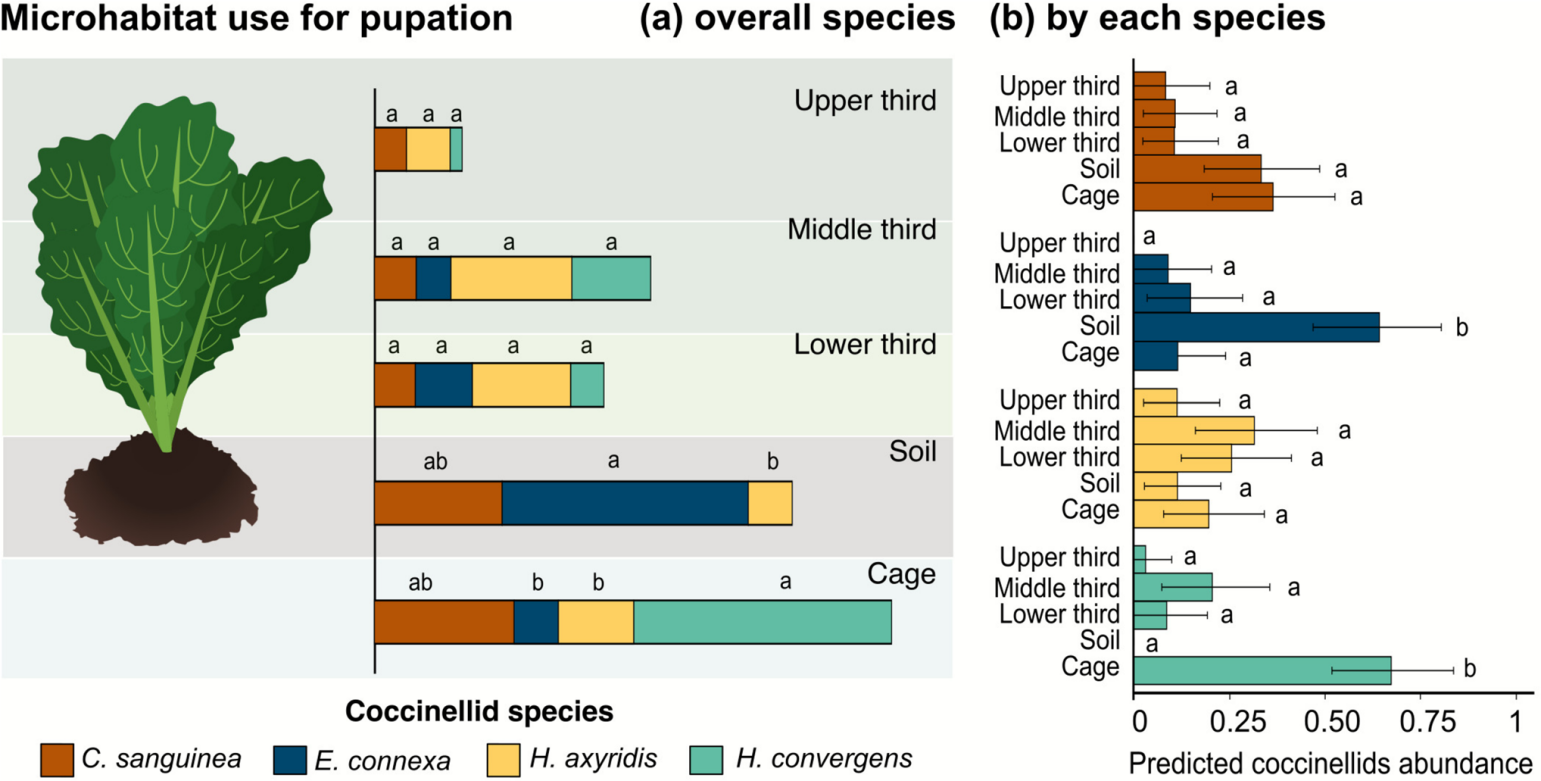
Predicted probabilities of microhabitat use for pupation by overall coccinellid species (**a**) and by each species (**b**), considering 95% confidence intervals based on 1000 bootstrap resamples. Different letters indicate significant differences among species within the same microhabitat (**a**) and by each species across different microhabitats (**b**)

The probability of microhabitat selection by coccinellid larvae for pupation also varied among species (χ^2^ = 69.5; d.f. = 12; *p* < 0.001). *Cycloneda sanguinea* and *H*. *axyridis* showed similar pupation probabilities across all microhabitats. *Eriopis connexa* was more likely to pupate on soil than in the other microhabitats. For *H*. *convergens*, the probability of pupation on the cage walls was greater than in any other microhabitat (Fig. 2). On the soil, *E*. *connexa* displayed a higher pupation probability than *H*. *axyridis*. Conversely, on the cage, *H*. *convergens* was more likely to pupate than both *E*. *connexa* and *H*. *axyridis* (Fig. 2).

**Fig. 3:**
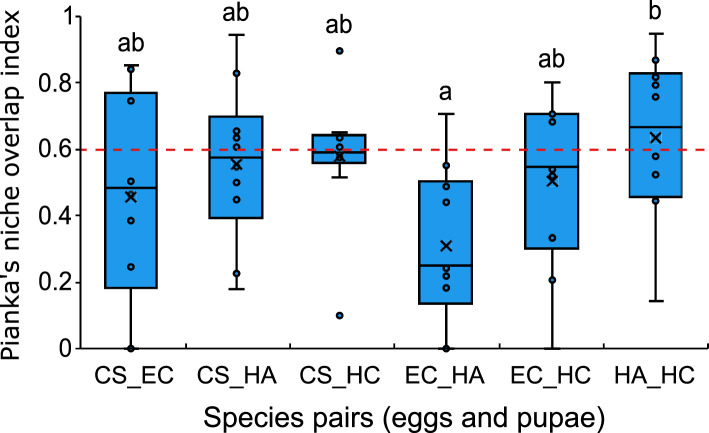
Pianka’s niche overlap index between coccinellid species pairs (eggs and pupae) using the lower, middle, and upper thirds of brassica crops and outside brassica plants in the cage experiment. Different letters indicate significant differences based on model contrast analysis (*p* < 0.05). CS = *Cycloneda sanguinea*, EC = *Eriopis connexa*, HA = *Harmonia axyridis*, HC = *Hippodamia convergens.* The horizontal line within the boxplot indicates the median and “*x*” denotes the mean. The dashed red line indicates where niche overlap reaches 60%

The species’ niche highly overlapped (> 60% for eggs + pupae) among species (χ^2^ = 15.5; d.f. = 5; *p* < 0.001). Nevertheless, the overall niche overlap for coccinellid eggs and pupae combined was not statistically different from the null models (Table 1), indicating that the observed niche overlap is attributable to chance. The pairwise comparisons showed that niche overlap between species included the 60% threshold (i.e., high niche overlap), except for *E*. *connexa* and *H*. *axyridis* (< 40%) (Fig. 3).

**Table 1:** Results from the null models for coccinellid species niche overlap in cage experiment and field sampling across different life stages

Data origin	Life-stage	Observed index	Expected (RA3) (10.000 iterations)				
			Mean	variance	Lower tail *P* = (obs < exp)	Upper tail *P* = (obs > exp)	Standardized effect size (SES)
Cage experiment	All	0.654	0.667	0.003	0.462	0.538	− 0.245
	Eggs	0.490	0.480	0.010	0.611	0.390	0.107
	Pupae	0.611	0.581	0.006	0.707	0.293	0.378
Field sampling	All	0.836	0.437	0.013	0.992	**0.009**	3.525
	Eggs	0.289	0.372	0.015	0.278	0.722	− 0.668
	Larvae	0.781	0.402	0.014	0.989	**0.011**	3.196
	Pupae	0.853	0.248	0.024	0.996	**0.016**	3.938
	Adults	0.758	0.366	0.016	0.990	**0.010**	3.130

The observed index corresponds to the average Pianka index calculated for all individuals in the cage experiments and field data. The expected mean and variance values were derived using the RA3 algorithm. Bold values indicate statistically significant results

### Habitat and microhabitat use and niche overlap in the field

Across all farms, we recorded an average of 107.1 ± 16.9 aphids per leaf on collards. The species observed were *Brevicoryne brassicae* (L.), *L. pseudobrassicae*, and *M. persicae*. On non-crop plants, only aphids from the genus *Uroleucon* were recorded. Although we did not estimate aphid density on non-crop plants, coccinellids were observed on plants hosting *Uroleucon* spp. at least 189 times across all farms. The mean abundance of coccinellids (larvae and adults) per farm ranged from 1.25 to 34.8 individuals per species. Considering all farms and only larvae and adults, *H. convergens* was the most abundant species (*n* = 1757), followed by *C. sanguinea* (*n* = 698), *E. connexa* (*n* = 616), and *H. axyridis* (*n* = 162). Egg abundance (including unidentified eggs) was highest on non-crop plants (*n* = 43), followed by the middle (*n* = 21), lower (*n* = 17), and upper (*n* = 5) plant thirds, and soil (*n* = 3). Larvae were also most abundant on non-crop plants (*n* = 508), followed by soil (*n* = 90), lower (*n* = 44), upper (*n* = 25), and middle (*n* = 15) thirds. Pupae (including unidentified) were mainly found on non-crop plants (*n* = 39), with only one on the soil and one in the middle third; none were found on the lower or upper thirds. Adults were most abundant on non-crop plants (*n* = 1662), followed by soil (*n* = 828), lower (*n* = 247), upper (*n* = 150), and middle (*n* = 114) thirds. A few eggs and pupae were identified to species level, including one *C. sanguinea* pupa on the soil (see Table S2 for details).

**Fig. 4:**
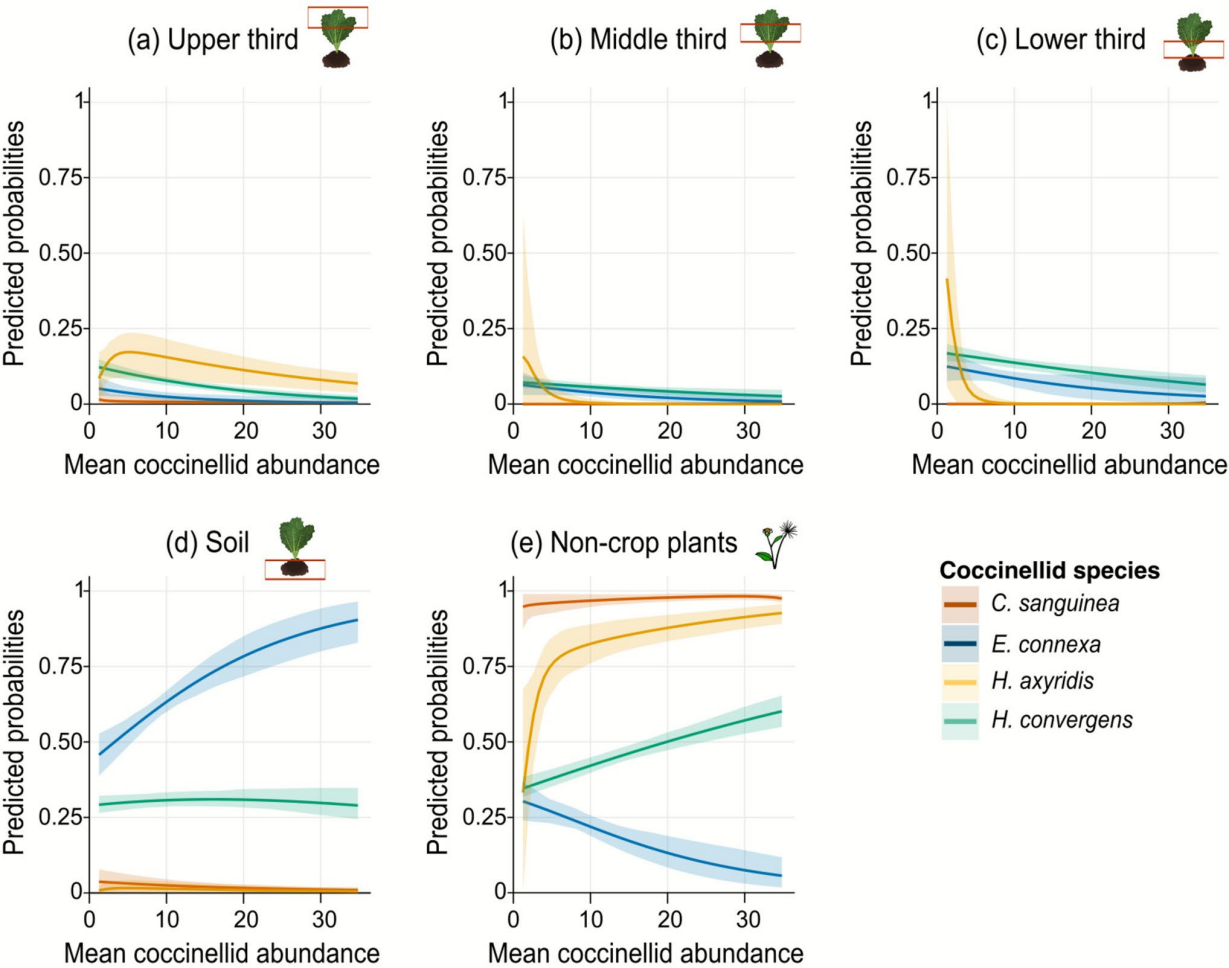
Predicted probabilities of habitat and microhabitat use (upper, middle, and lower thirds; soil; and non-crop plants) by each coccinellid species at different mean coccinellid abundance per farm. Shaded areas indicate 95% bootstrap confidence intervals (1000 resamples)

The probability of coccinellid occurrence (larvae and adults) at habitat and microhabitat scales was influenced by species identity (*χ*^2^ = 885.8; *d.f*. = 12; *p* < 0.001), the mean coccinellid abundance in farms (*χ*^2^ = 72.9; *d.f*. = 4; *p* < 0.001), and the interaction between these variables (*χ*^2^ = 52.2; *d.f*. = 12; *p* < 0.001). This demonstrates that species exhibit specific site preferences modulated by coccinellid abundance. For the thirds of brassica plants, the predicted probabilities of species occurrence highly overlapped (predicted probability of occurrence below 25%), with a tendency to decrease when coccinellid abundance on farms was higher (Fig. 4a–c). The probability of *E*. *connexa* using the soil was higher than for other species, ranging over 75% when mean coccinellid abundance in farms increased (Fig. 4d). Also on the soil, the probability of *H*. *convergens*’ presence was higher than that of *C*. *sanguinea* and *H*. *axyridis* (both reaching zero). Despite the increase in mean coccinellid abundance, the probability of *H*. *convergens* occurring on the soil remained constant, slightly above 25% (Fig. 4d).

The probability of species using non-crop plants varied notably. *Cycloneda sanguinea* had the highest probability (close to 100%), regardless of mean coccinellid abundance (Fig. 4e). For *H*. *axyridis*, this probability rose from under 50% to nearly 100% as coccinellid abundance increased. *Hippodamia convergens* also exhibited a consistent increase in the probability of using non-crop plants as coccinellid abundance increased, reaching approximately 60%. In contrast, *E*. *connexa* was more likely to use non-crop plants when coccinellid abundance was low (about 25% at fewer than the mean of 10 individuals), but this probability declined sharply with increasing abundance, approaching zero (Fig. 4d).

**Fig. 5:**
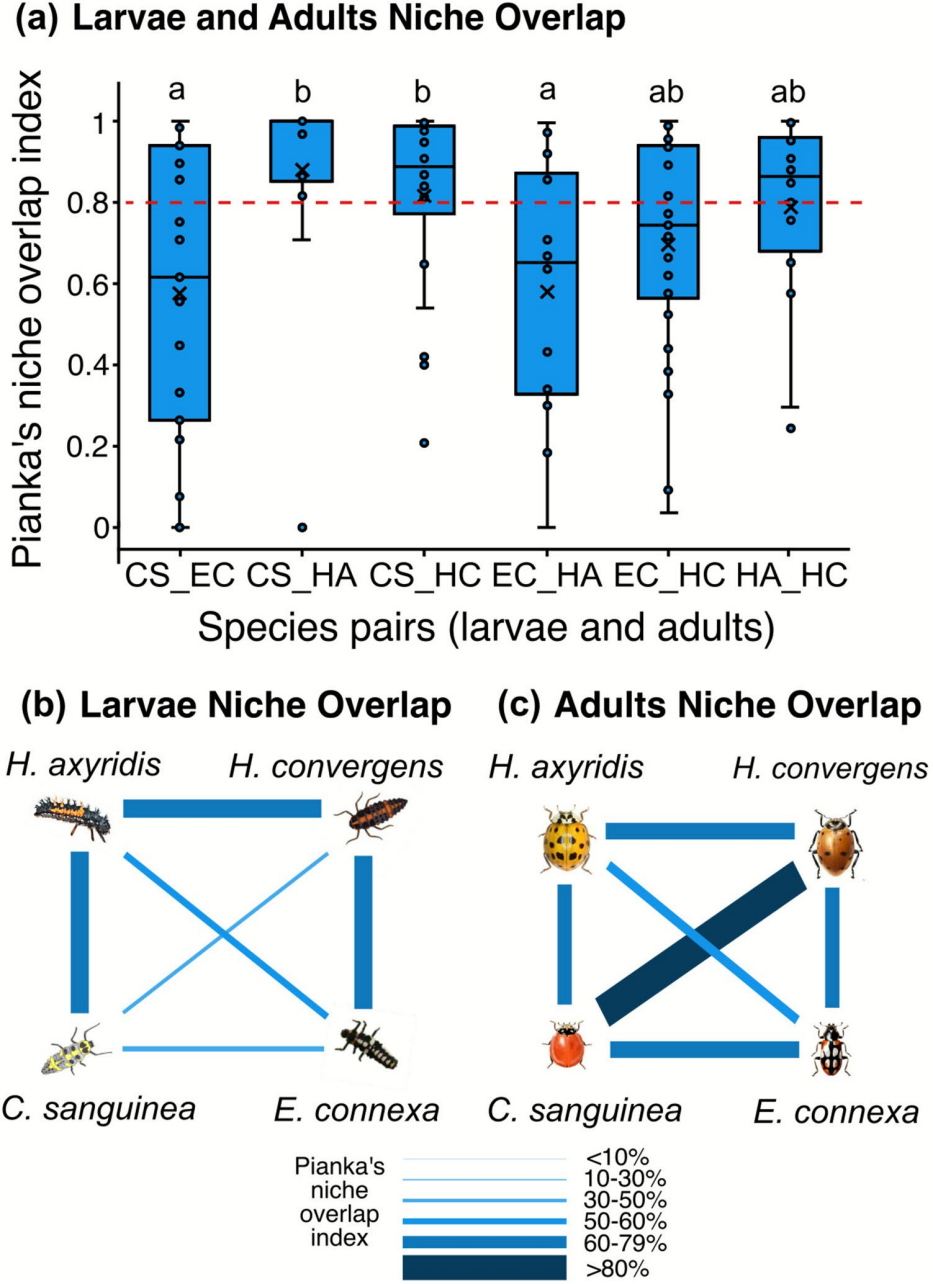
**a** Pianka’s niche overlap index between coccinellid species pairs (larvae and adults) using the lower, middle, and upper thirds of brassica crops and the non-crop plants in the field, and average Pianka’s niche overlap index between larvae (**b**) and adults (**c**) species pairs. Different letter combinations indicate significance determined by model contrast analysis (*p* < 0.05). The horizontal line within the boxplot indicates the median, and the “*x*” the mean. Dashed red line indicates where niche overlap reaches 80%. Cs = *Cycloneda sanguinea*, Ec = *Eriopis connexa*, Ha = *Harmonia axyridis*, Hc = *Hippodamia convergens*

The niche overlap was higher (> 80%) between *C*. *sanguinea* and both *H*. *axyridis* and *H*. *convergens*, and lower (< 60%) between *C*. *sanguinea* and *E*. connexa, as well as between *E*. *connexa* and *H*. *axyridis*, indicating that niche overlap varied among pairs of species (*χ*^2^ = 25.1; *d.f*. = 5; *p* < 0.001) (Fig. 5a). The niche overlap varied across life stages, with larvae showing higher overlap for *E*. *connexa* and *H*. *convergens* (79%) and lower for *E*. *connexa* and *C. sanguinea* (37%) (Fig. 5b). Adults had a higher overlap for *C*. *sanguinea* and *H*. *convergens* (84%) and lower for *E*. *connexa* and *H*. *axyridis* (54%) (Fig. 5c). Coccinellids’ niche overlap was 83% for all life stages, 28% for eggs, 78% for larvae, 85% for pupae, and 75% for adults. It differed statistically from chance relative to null models, except for eggs (Table 1; Fig. S1).

## Discussion

The aphidophagous coccinellids *C. sanguinea, E. connexa, H. convergens*, and *H. axyridis* exhibited distinct patterns of habitat and microhabitat use across life stages, with niche overlap varying especially during the mobile stages (larvae and adults). Species-specific traits related to space use influenced individual response to the increased abundance of con- and heterospecifics on farms. Non-crop plants may enable species to co-occur at the community level by altering niche overlap across life stages. Therefore, even under conditions of potentially strong competition, species can co-occur in spatially heterogeneous environments due to behavioral plasticity in context-dependent interactions. These behaviors include unexpected tendencies in some species to lay eggs and pupate on the soil surface, especially *E. connexa*.

### Spatial segregation of habitat and microhabitat use across different life stages

All coccinellid species studied oviposited on collard plants, reflecting females’ active search for aphid patches for their offspring, as larvae have limited mobility (Seagraves 2009). However, oviposition behavior varied among species. While *H. axyridis* and *C. sanguinea* readily deposited eggs within each of the collard sections, *H. convergens* preferred to oviposit in the lower third of the collards and on the soil. *Eriopis connexa* exhibited an unexpected tendency to lay eggs on the soil surface. Although *Coccinella septempunctata* (L.) (Coleoptera: Coccinellidae) was previously reported to oviposit on the soil (Ferran et al. 1989), this is the first record of this behavior for *E. connexa*. These differences may reflect behavioral trade-offs involving prey access (Seagraves 2009), predation risk (including intraguild predation) (Hodek et al. 2012), and bet-hedging strategies (Evans 2003), influencing species-specific oviposition strategies.

Pupation site choice also varied among species, but the mechanisms underlying these changes differ from those for oviposition site selection. Pupation site selection may be more conditioned to avoid intraguild predation (Lucas et al. 2000) and reduce exposure to parasitoids (Togni et al. 2015; Paula et al. 2021). *Harmonia* axyridis prefers to pupate on the plant, possibly saving energy by not moving to other areas (Ferran and Dixon 1993). Conversely, the other species employ specific strategies to minimize exposure to natural enemies (mostly pupae) or competitors (larvae, adults, and oviposition sites) (Lima and Dill 1990). *Eriopis connexa* predominantly pupates on the soil, where other coccinellid species usually do not forage, and *H. convergens* tends to avoid the plants with aphids and preferentially uses alternative sites above the soil. However, *C. sanguinea* dilutes the exposure risk during the pupal stage using soil and alternative sites above the soil equally. Interestingly, this is the first report of all three species pupating on the soil. Therefore, pupation site choice may be an innate behavior that has been selected for in this guild in a risky environment with high exposure to natural enemies.

Under field conditions, the use of plant strata by mobile stages depended on coccinellid abundance on farms, possibly reflecting varying levels of competition, since both intra- and interspecific competition are density-dependent (Amarasekare 2003b). However, in our study, these effects may be restricted to the plant level or at most to the patch level. The middle third of collard plants was used less frequently and in similar ways by most species. In the upper third, *H. axyridis* was more frequent, particularly when coccinellid abundance was low. In contrast, in the lower third, *H. axyridis* was replaced by *H. convergens* and *E. connexa* as coccinellid abundance increased, possibly due to interference competition (Amarasekare 2003b). The increased presence of these species on the lower third and on the soil suggests spatial shifts due to heterospecific avoidance or competitive displacement (Lucas et al. 2000; Reitz and Trumble 2002) and fine-scale microhabitat partitioning among species. In contrast, *C. sanguinea* was more closely associated with non-crop plants due to its ability to exploit prey such as *Uroleucon* spp. (Hemiptera: Aphididae) (Sicsú et al. 2020), associated with avoidance behavior or displacement by dominant species in the crop area.

On the soil surface, *E. connexa* was the only species whose occurrence increased with coccinellid abundance. This result reinforces its role as a ground-dwelling predator that opportunistically forages on plants. This species may prey on aphids dislodged from the plant by other predators foraging on it, as demonstrated for other ground-foraging beetles (e.g., Losey and Denno 1998). However, the use of soil as an oviposition or pupation site may be suboptimal because eggs and pupae are immobile and vulnerable to epigeal predators. This may explain why eggs on the soil were less frequent under field conditions than expected based on the cage experiment.

Despite being a superior competitor (Andow et al. 2023), *H. convergens* became less abundant on the soil as coccinellid abundance increased, possibly because *E. connexa* outcompeted it. At the same time, *C. sanguinea* and *H. axyridis* rarely used the soil. This may be due to their reliance on aphids and plant-provided food (Amaral et al. 2013), and their presumably limited ability to exploit fallen aphids on the soil, where *E. connexa* thrives. These findings highlight how microhabitat specialization and context-dependent presumed negative interactions shape space use in aphidophagous coccinellids.

### Non-crop plants may mediate species co-occurrence at the community level

On non-crop plants, coccinellid species exhibited opposite trends to those observed on the soil. *Eriopis connexa* had the lowest probability of occurrence in these areas among all species, and its use declined with increasing coccinellid abundance. The opposite was observed for *H. convergens,* probably due to its generalist feeding habits (Michaud 2018) and flexibility in habitat use across life stages. Nevertheless, all species used non-crop plants to some extent, benefiting from habitat structural complexity, which enhances individuals’ movement, favors niche partitioning among intraguild predators (Da Silva et al. 2022), and provides shelter for different life stages (Togni et al. 2016).

Notably, *C. sanguinea* was consistently associated with non-crop plants irrespective of coccinellid abundance. This may be because the species either avoids or is excluded from collards, or does not primarily feed on the aphids on this plant s. Although *C. sanguinea* individuals can develop on *Brevicoryne brassicae*, they prefer or are constrained to non-crop habitats due to competitive exclusion or prey specialization. While the genus *Uroleucon* is widespread, several species are native to the Neotropical and Nearctic regions, primarily occurring on Asteraceae and Campanulaceae plants (de Carvalho et al. 1998). In our study system, *Uroleucon* species were found mainly in non-crop areas on *Bidens pilosa* (Asteraceae), and this plant species can increase *C. sanguinea*’s fitness, whereas *B. brassicae* is a less suitable prey species (Sicsú et al. 2020; Andow et al. 2023). Therefore, non-crop resources may enable native and competitively inferior species to co-occur in the community (Fonseca et al. 2017).

*Harmonia axyridis*, an exotic species, might not use native non-crop plant resources (aphids, pollen, and nectar) (Amaral et al. 2013), possibly relying mostly on crop aphids like *B. brassicae* (Sicsú et al. 2020). However, its increased use of non-crop plants under high coccinellid abundance suggests that superior competitors, such as *H. convergens* (Andow et al. 2023), displace it. However, on non-crop plants, *C. sanguinea* can exploit resources that *H. axyridis* cannot (Amaral et al. 2013). This may explain its lower abundance in the studied farms and its failure to successfully invade organic crops in this region (Paula et al. 2021; Andow et al. 2023).

These results point to species-specific patterns of habitat use shaped by innate preferences, prey availability, and possibly by predatory interactions (Reitz and Trumble 2002). The use of non-prey food resources (Fonseca et al. 2017; Venzon et al. 2019) and the unexpected oviposition on soil by some species suggest behavioral plasticity. Habitat and microhabitat use in aphidophagous coccinellids is both species- and stage-dependent but may shift in response to interspecific competition and intraguild predation on specific life stages (mostly larvae and adults), reinforcing the ecological value of behavioral plasticity (Seagraves 2009).

### Coccinellids’ niche overlap changes throughout their life stages

Combined, our experiments revealed that mobile stages of coccinellids (larvae and adults) exhibited greater niche overlap than immobile stages (eggs and pupae), which remained spatially segregated. Immobile stages are fixed in place until hatching/emergence. Therefore, safe sites from natural enemies may result in a higher spatial segregation to avoid natural enemy spillover due to density-dependent effects (Paula et al. 2021) and to reduce detectability by intraguild predators and competitors (Rocca et al. 2022).

The high overlap between *H. axyridis* and *C. sanguinea* suggests that both species occur at the microhabitat and patch levels. In contrast, functional asymmetries appear to mediate coexistence in species that highly overlap their spatial niche. *Hippodamia convergens* exhibits higher prey capture efficiency in brassica crops (Andow et al. 2023), reinforcing its dominance along a key niche axis. However, *E. connexa* may be a more efficient ground-foraging predator than *H. convergens*, thereby stabilizing coexistence (Amarasekare 2003b). Similarly, while *C. sanguinea* and *H. axyridis* also overlapped with *H. convergens*, this overlap is reduced for *C. sanguinea* during the larval stage.

Our results suggest that aphidophagous coccinellid communities are structured by differences in spatial niche overlap varying across different life stages. Although spatial niche overlap among the immobile stages did not differ from random, overlap among mobile stages was non-random and structured, thereby reinforcing the relevance of behavioral plasticity in mediating spatial partitioning. While we measured niche based on spatial distribution, niches are inherently multidimensional, and factors such as prey identity, phenology, or temporal dynamics may yield different results. Despite this limitation, our findings support previous evidence that *H. axyridis*, although a highly invasive exotic species (Martins et al. 2009; Roy et al. 2016), did not establish in the organic systems in central Brazil because other species may outcompete it due to more efficient resource use (Andow et al. 2023), especially plant-provided food (Amaral et al. 2013), associated with a high pressure of native parasitoids (Paula et al. 2021).

## Conclusions

We demonstrated that aphidophagous coccinellids coexist in agroecosystems through spatial niche partitioning, which is modulated by environmental heterogeneity and life-stage differences in habitat demands. Habitat and microhabitat use varied among species and life stages, influenced by both innate behaviors and plastic responses to density-dependent effects of coccinellid abundance. We propose that the species we studied may be functionally classified based on their behavioral responses across life stages. *Hippodamia convergens* acted as a risk-tolerant species that thrives in high-density crop habitats where prey are abundant, but competition is more likely to occur when aphid densities are low. At the same time, when aphid densities are high, intraguild predation may occur due to density-dependent effects that attract more individuals to the area. By contrast, *C. sanguinea* acted as a risk-avoider, confined to non-crop vegetation probably due to lower interspecific competition and greater access to less abundant (mostly native) and more diverse resources, at the cost of reduced access to high-prey-density areas. *Eriopis connexa* acts as a niche-fidelity specialist, occupying less competitive habitats and reinforcing its soil-foraging specialization as coccinellid abundance increases, potentially reducing predation and competition over time. Notably, this species consistently foraged, oviposited, and pupated on soil, likely acting as a soil specialist. Although *H. axyridis* may be classified as a risk-tolerant species in areas where it has successfully invaded, in our study system, it behaves as a conditional risk-avoider. It thrives in suitable habitats with presumed low interspecific competition but declines when displaced to less favorable ones, due to its strong dependence on aphids and limited resource-use flexibility in the invaded area. Those classifications could be broadened and tested on other aphidophagous coccinellids and validated on other systems. Our findings suggest that trade-offs in competitive ability, habitat use, and exposure to risk across life stages shape divergent ecological strategies of aphidophagous coccinellids and stabilize their coexistence.

## Supplementary Information

Below is the link to the electronic supplementary material.

Supplementary file1 (DOCX 350 KB)

## Data Availability

Data will be made publicly available in the public repository Figshare, upon acceptance of the manuscript.
