# Supplementary material for: Life-stage niche partitioning and functional strategies promote predatory coccinellids’ co-occurrence
Source: Oecologia. 2026 Jan 31;208(2):26. doi: 10.1007/s00442-026-05866-w (PMC12858511; doi:10.1007/s00442-026-05866-w)
Supplement: Supplementary file 1 — Supplementary file1 (DOCX 350 KB) [file 442_2026_5866_MOESM1_ESM.docx]

**Supplementary Information**

**Life-stage niche partitioning and functional strategies promote predatory coccinellids’ co-occurrence**

Ana Claudia da Silva^1^, Débora P. Pires^2^, David Andow^3^, Patricia S. Sujii^4^, Nícholas F. Camargo^4^, Pedro H. B. Togni^4,*^

^1^ Programa de Pós-graduação em Ecologia, Instituto de Ciências Biológicas, Universidade de Brasília (UnB), Brasília, DF, Brazil

^2^ Embrapa Recursos Genéticos e Biotecnologia, Brasília, DF, Brazil

^3^ Department of Applied Ecology, North Carolina State University, Raleigh, North Carolina, USA

^4^ Departamento de Ecologia, Instituto de Ciências Biológicas, Universidade de Brasília (UnB), Brasília, DF, Brazil

*Corresponding author: Departamento de Ecologia, Universidade de Brasília (UnB), Campus Darcy Ribeiro, 70910-900, Asa Norte, Brasília, Distrito Federal, Brazil. Email pedrotogni@unb.br Phone: +55 61 3107 2993


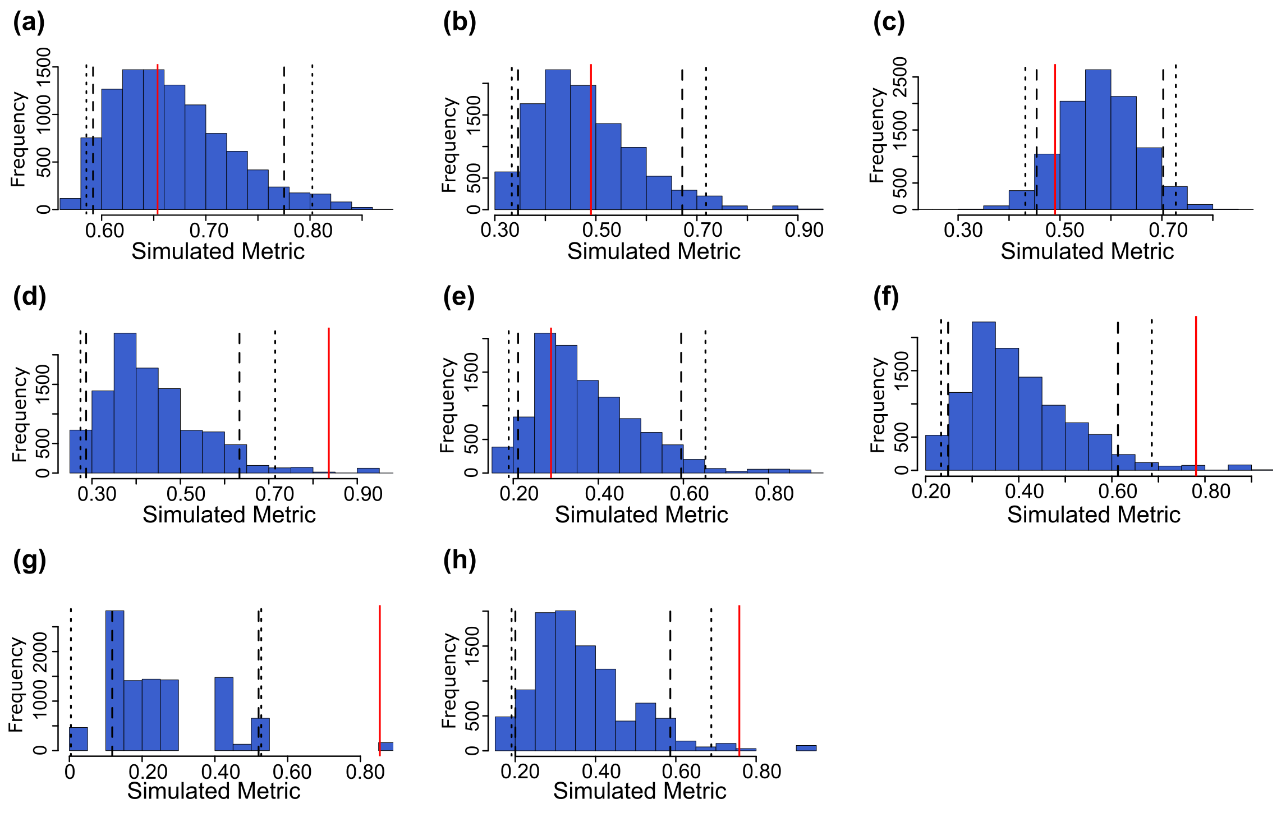


**Fig. S1.** Expected values of niche overlap (blue bars) of coccinellids communities based on Pianka index. Each graph represents all life stages (a), only eggs (b) and only pupae (c) for the cage experiment. All life stages (d), only eggs (e), only larvae (f), only pupae (g) and only adults (h) for the field experiments. The vertical red lines represent the observed niche values, the long-dash lines show the one-tailed 95% limits, and the short-dash lines represent the two-tailed 95% limits.

**Table S1**. Models and parameters from multinomial logistic regression models on the effects of coccinellid species, coccinellid life stage, and mean coccinellid abundance in the habitat and micro-habitat selection by coccinellids (the response variable used for all models) on 42 organic brassica-producing farms in the Brazilian Federal District between 2020 and 2023. Bold numbers indicate statistically significant differences at the 0.05 significance level.

| **Model** | **Explanatory variables** | **logLink** | **AICc** | **ΔAICc** | **Relative weight** | **χ²** | **d.f.** | **p.value** |
| --- | --- | --- | --- | --- | --- | --- | --- | --- |
| 4 | **Coccinellid species** | -3186.66 | 6437.99 | 0 | 0.666 | 885.76 | 12 | **<0.001** |
|  | **Mean coccinellid abundance** |  |  |  |  | 72.88 | 4 | **<0.001** |
|  | **Interaction** |  |  |  |  | 52.17 | 12 | **<0.001** |
| 7 | **Coccinellid species** | -3183.27 | 6439.37 | 1.38 | 0.334 | 856.66 | 12 | **<0.001** |
|  | **Mean coccinellid abundance** |  |  |  |  | 73.82 | 4 | **<0.001** |
|  | Coccinellid life stage |  |  |  |  | 6.79 | 4 | 0.147 |
|  | **Coccinellid species: mean abundance** |  |  |  |  | 51.17 | 12 | **<0.001** |
| 3 | **Coccinellid species** | -3212.75 | 6465.76 | 27.77 | 0 | 885.76 | 12 | **<0.001** |
|  | **Mean coccinellid abundance** |  |  |  |  | 72.88 | 4 | **<0.001** |
| 2 | **Coccinellid species** | -3208.85 | 6530.55 | 69.89 | 0 | 1223.7 | 12 | **<0.001** |
| 5 | **Coccinellid species** | -3245.77 | 6531.79 | 71.14 | 0 | 1110.20 | 12 | **<0.001** |
|  | Coccinellid life stage |  |  |  |  | 6.85 | 4 | 0.144 |
| 6 | **Coccinellid species** | -3242.19 | 6549.04 | 88.38 | 0 | 1110.20 | 12 | **<0.001** |
|  | Coccinellid life stage |  |  |  |  | 6.85 | 4 | 0.144 |
|  | Interaction |  |  |  |  | 7.16 | 12 | 0.847 |
|  |  |  |  |  |  |  |  |  |
| 1 | ~ 1 | -3861.02 | 7730.05 | 1369.58 | 0 | N.A. | N.A. | N.A. |

**Table S2.** Abundance of egg masses and pupae recorded across 42 organic brassica-producing farms in the Brazilian Federal District between 2020 and 2023, found on brassica plant thirds (upper, middle, and lower), soil, and non-crop plants.

| **Egg masses** | | | | | | |
| --- | --- | --- | --- | --- | --- | --- |
| **Site** | ***Cycloneda sanguinea*** | ***Eriopis connexa*** | ***Harmonia axyridis*** | ***Hippodamia convergens*** | **Unknown** | **Total** |
| Upper third | 1 | 1 | 0 | 0 | 3 | 5 |
| Middle third | 4 | 0 | 0 | 2 | 15 | 21 |
| Lower third | 0 | 0 | 0 | 5 | 12 | 17 |
| Soil | 0 | 0 | 0 | 0 | 3 | 3 |
| Non-crop plants | 5 | 0 | 0 | 0 | 38 | 43 |
| **Pupae** | | | | | | |
| Upper third | 0 | 0 | 0 | 0 | 0 | 0 |
| Middle third | 0 | 0 | 0 | 0 | 1 | 1 |
| Lower third | 0 | 0 | 0 | 0 | 0 | 0 |
| Soil | 1 | 0 | 0 | 0 | 0 | 1 |
| Non-crop plants | 1 | 0 | 1 | 4 | 33 | 39 |
